# Supplementary material for: Self-preservation strategy for approaching global warming targets in the post-Paris Agreement era
Source: Nat Commun. 2020 Apr 14;11:1624. doi: 10.1038/s41467-020-15453-z (PMC7156390; doi:10.1038/s41467-020-15453-z)
Supplement: Supplementary file 1 — Supplementary Information [file 41467_2020_15453_MOESM1_ESM.pdf]

Supplementary Information

Self-preservation strategy for approaching global warming targets in the post-Paris Agreement era

Wei et al.

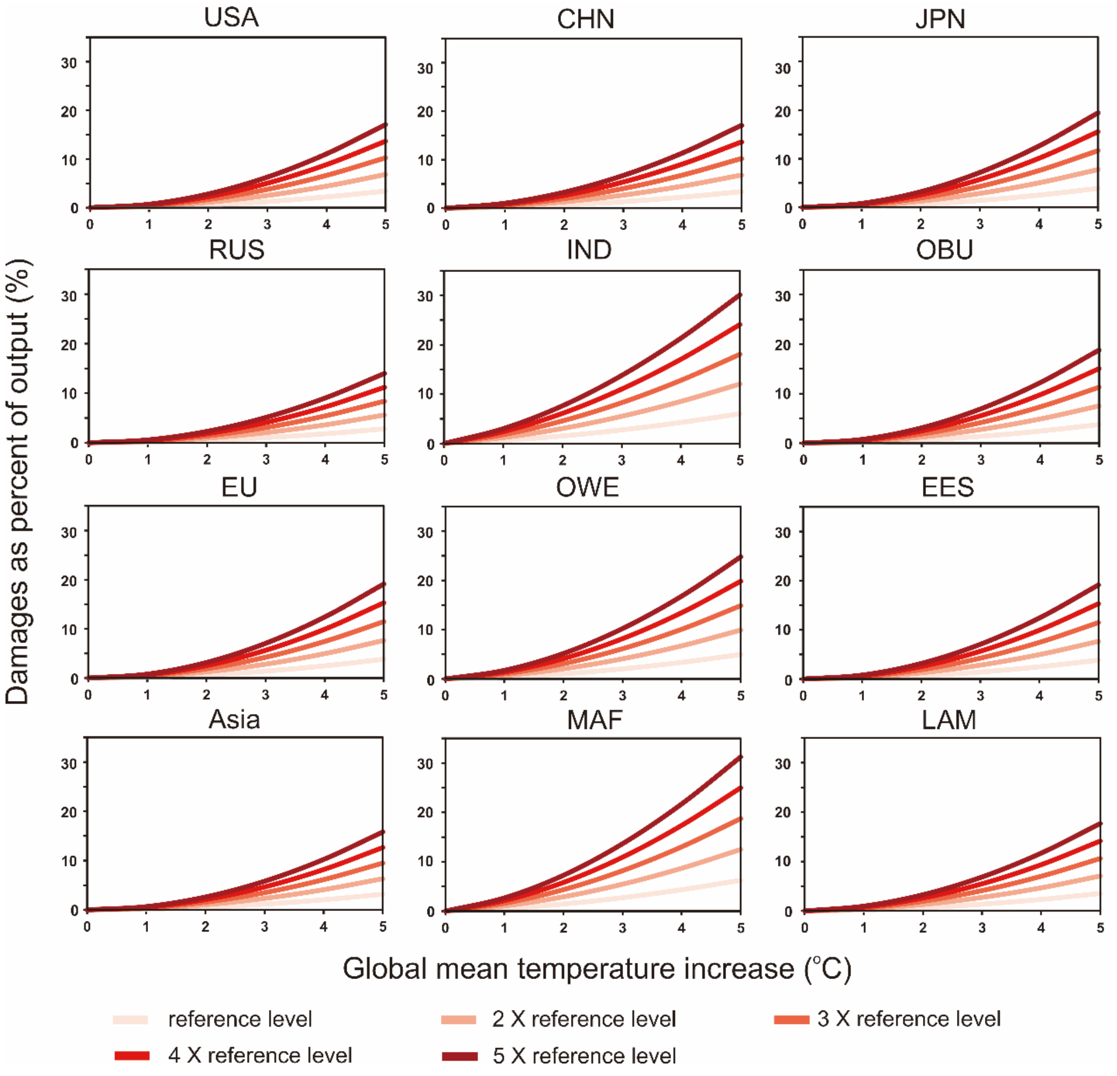

Supplementary Figure 1| The climate damage associated with different temperature rise. The values reported in the reference 19 is set as the reference level, which show a climate damage being approximately 1.6% of GDP at a 2.62 °C warming in 2100.

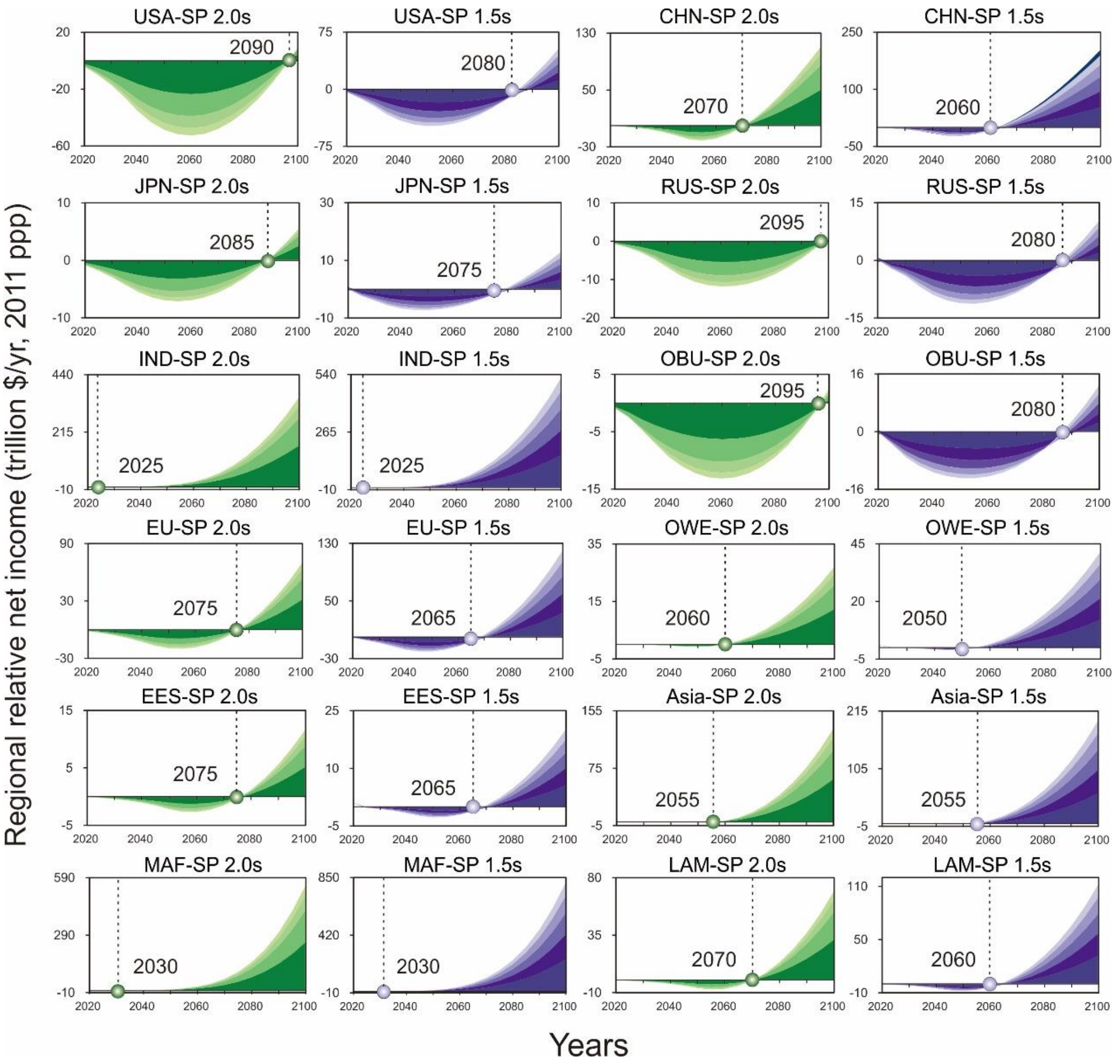

Supplementary Figure 2| Regional relative net income between policy-as-usual scenario following the current reduction efforts and SP 2.0s or SP 1.5s. Unit, trillion dollars per year. The circle callout represents the earliest turnaround year when cumulative relative benefits outweigh cumulative relative costs in the scenario group. The four green lines and five purple lines are the relative net income of the SP 2.0s and SP 1.5s, respectively.

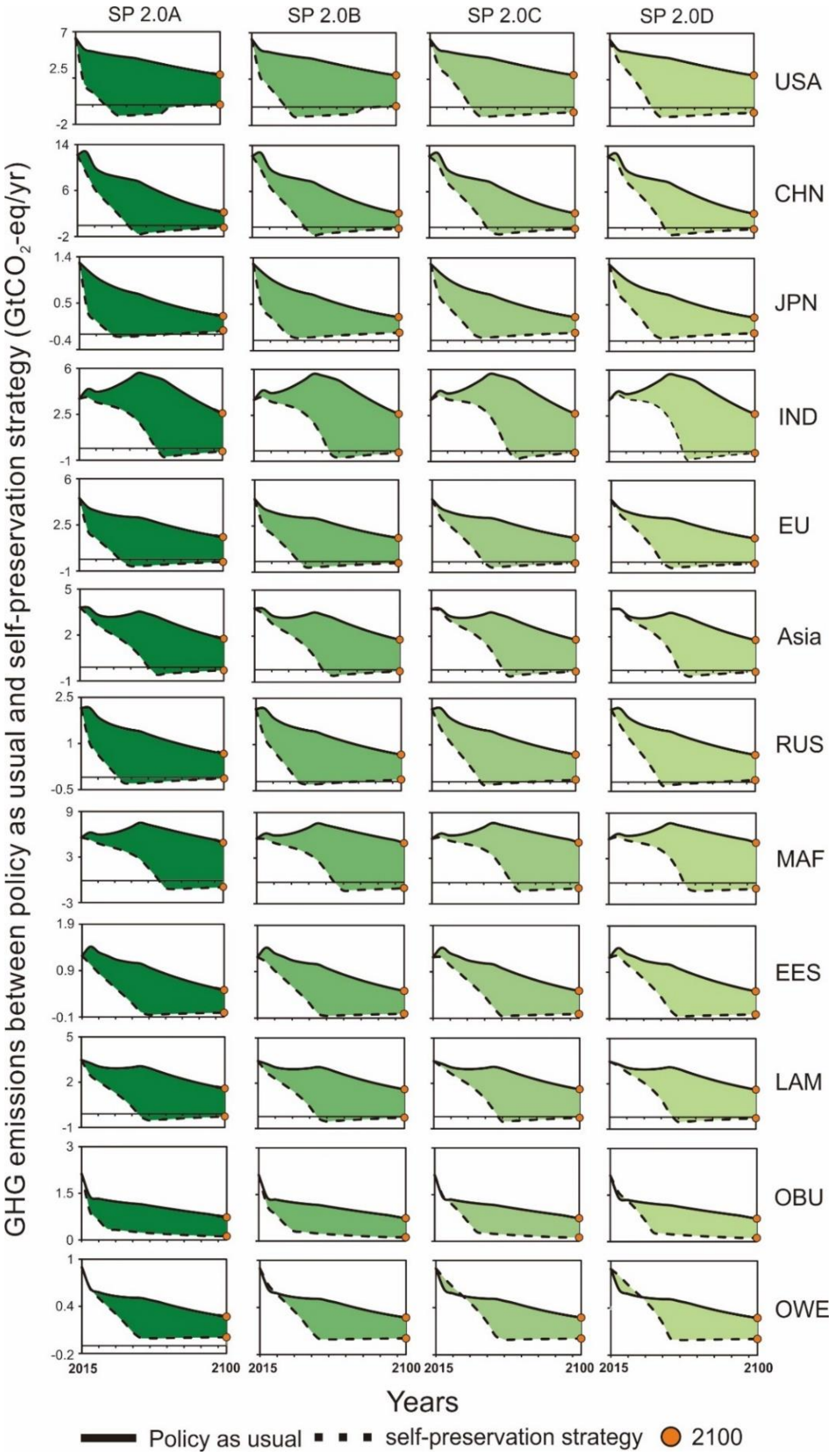

Supplementary Figure 3| Regional emissions gap between policy-as-usual scenario following the current reduction efforts and SP 2.0s. The solid and dashed lines represent the GHG emissions paths of the policy-as usual-scenario following the current NDCs and SP 2.0s, respectively.

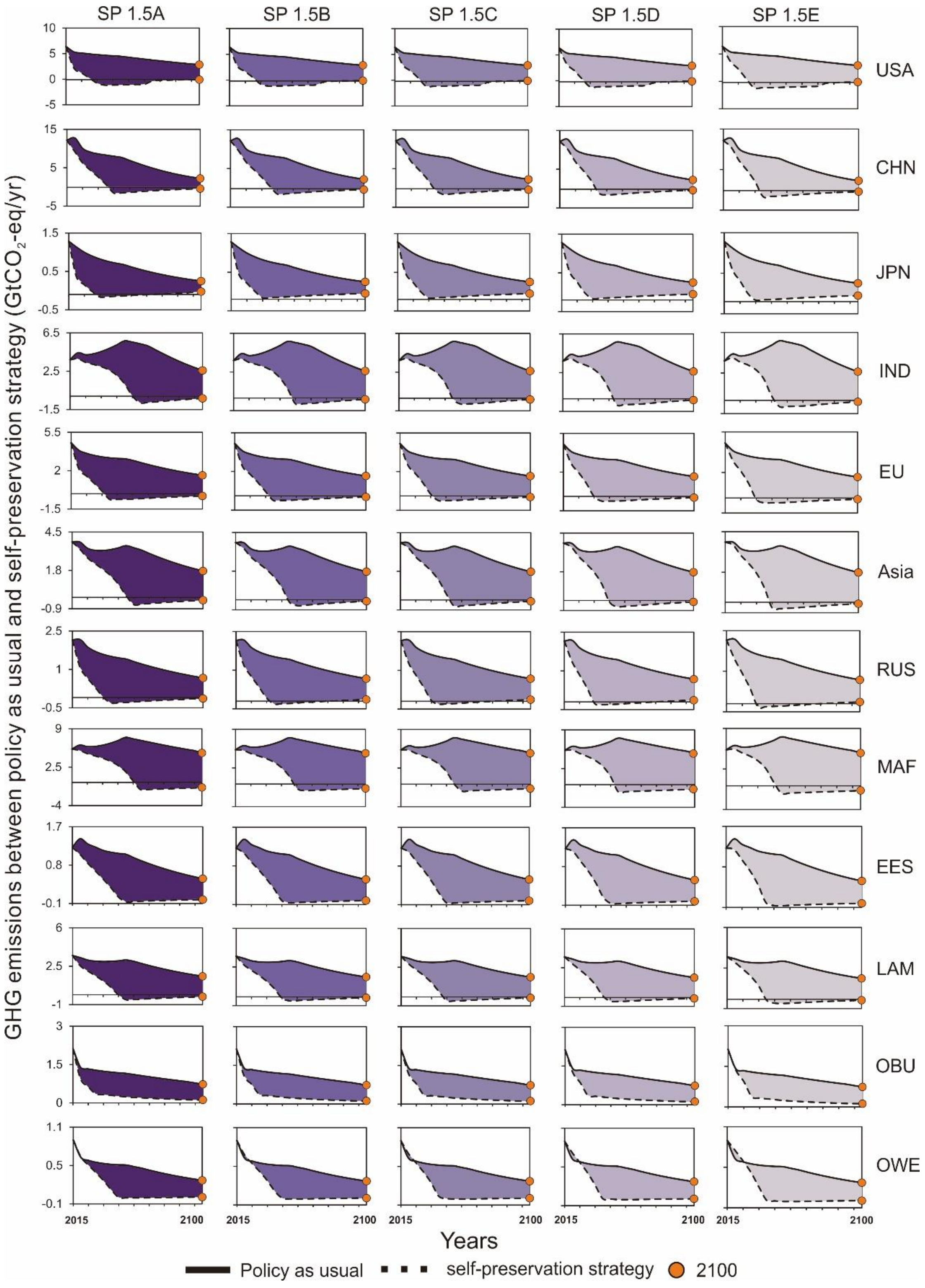

Supplementary Figure 4| Regional emissions gap between policy-as-usual scenario following the current reduction efforts and SP 1.5s. The solid and dashed lines represent the GHG emissions paths of the policy-a- usual scenario following the current NDCs and SP 1.5s, respectively.

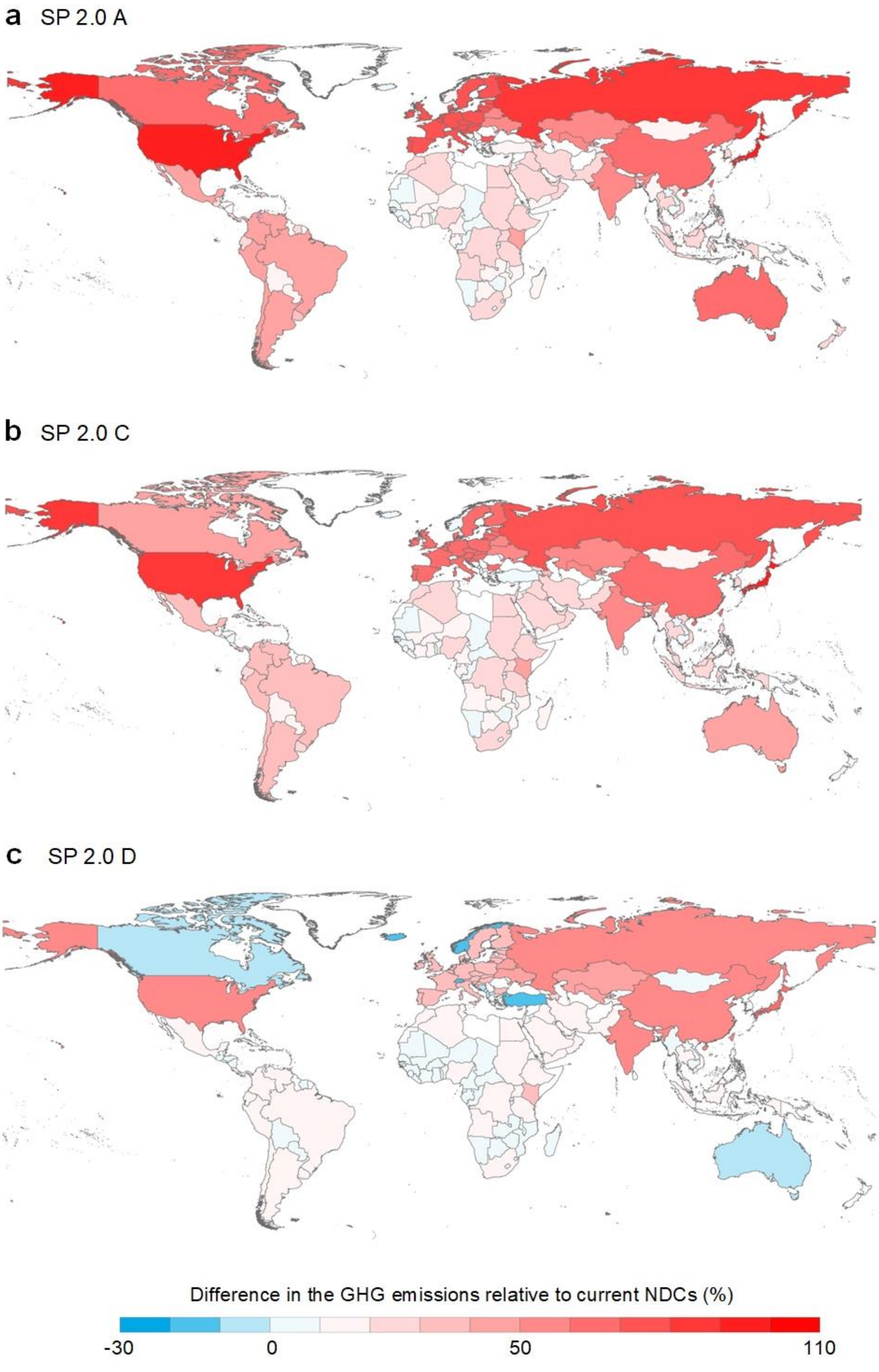

Supplementary Figure 5 | GHG emissions gap for each country under the SP 2.0s. The gap is the difference in the GHG emissions between current NDCs and the rest of SP 2.0s, and the positive value indicates the required improvement in GHG emissions reduction.

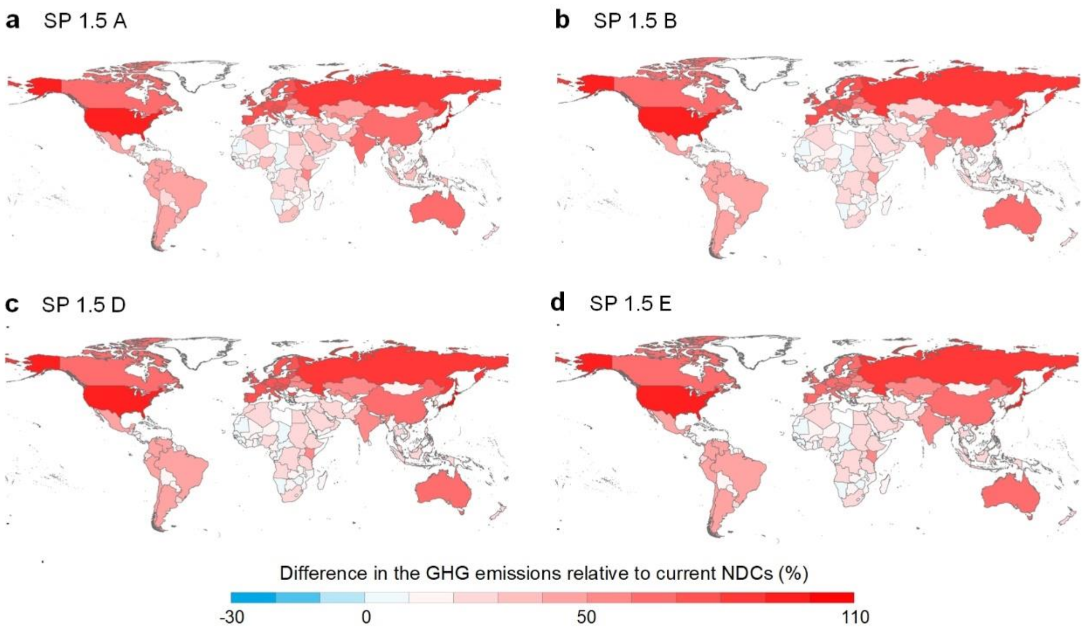

Supplementary Figure 6| GHG emissions gap for each country under the SP 1.5s. The gap is the difference in the GHG emissions between current NDCs and the rest of SP 1.5s, and the positive value indicates the required improvement in GHG emissions reduction.

Supplementary Table 1| Social weights for each region.

Region	Equity Principles				Average weight
	Ability to pay	Equal per capita allocation	Grandfathering	Historical responsibility	
USA	0.02	0.04	0.19	0.02	0.07
CHN	0.09	0.19	0.13	0.02	0.11
JPN	0.03	0.02	0.04	0.11	0.05
RUS	0.05	0.02	0.10	0.07	0.06
IND	0.20	0.18	0.05	0.07	0.12
OBU	0.03	0.01	0.04	0.12	0.05
EU	0.03	0.07	0.18	0.03	0.08
OWE	0.04	0.02	0.01	0.26	0.08
EES	0.11	0.02	0.06	0.14	0.08
Asia	0.13	0.15	0.06	0.06	0.10
MAF	0.18	0.19	0.08	0.04	0.12
LAM	0.09	0.08	0.07	0.06	0.07

Supplementary Table 2| Improved actions for achieving the 2 °C target for each country.

			$MtCO_2$-eq	Improved proportion (%)					
No.	ISO	Country	Current NDC	SP2.0A	SP2.0B	SP2.0C	SP2.0D	Region	NDC Type
1	JPN	Japan	1019.97	99.20	91.56	71.37	64.41	JPN	Base year
2	USA	United States	5096.13	94.50	83.61	61.45	53.01	USA	Base year
3	RUS	Russian	2265.35	85.45	78.61	64.37	59.50	RUS	Base year
4	EU	EU	3409.51	72.81	61.53	40.79	33.87	EU	Base year
5	CAN	Canada	520.41	66.08	45.84	6.94	-5.92	OBU	Base year
6	CHN	China	13288.86	65.88	61.25	53.21	50.62	CHN	Intensity
7	AUS	Australia	411.95	65.71	45.58	6.90	-5.89	OBU	Base year
8	IND	India	8071.76	59.66	58.00	55.08	54.16	IND	Intensity
9	UKR	Ukraine	527.69	57.94	52.91	44.15	41.31	EES	Base year
10	KAZ	Kazakhstan	318.11	57.88	52.85	44.10	41.26	EES	Base year
11	UZB	Uzbekistan	491.87	57.34	52.36	43.69	40.88	EES	Intensity
12	BLR	Belarus	64.16	56.62	51.70	43.14	40.37	EES	Base year
13	BRA	Brazil	633.18	47.35	38.95	24.32	19.58	LAM	Base year
14	MEX	Mexico	638.21	46.98	38.65	24.13	19.43	LAM	BAU
15	ARG	Argentina	483.00	45.90	37.77	23.58	18.99	LAM	BAU
16	VEN	Venezuela,	362.52	45.06	37.07	23.14	18.64	LAM	BAU

17	COL	Colombia	179.97	45.00	37.02	23.11	18.61	LAM	BAU
18	CHL	Chile	185.69	44.49	36.60	22.85	18.40	LAM	Intensity
19	PER	Peru	93.67	44.34	36.48	22.77	18.34	LAM	BAU
20	AZE	Azerbaijan	60.60	41.18	37.61	31.38	29.36	EES	Base year
21	TTO	Trinidad and Tobago	28.96	34.23	28.16	17.58	14.16	LAM	BAU
22	URY	Uruguay	36.17	30.29	24.92	15.56	12.53	LAM	Actions
23	NGA	Nigeria	610.31	29.82	25.94	19.20	17.03	MAF	BAU
24	IRN	Iran	826.85	29.82	25.94	19.20	17.03	MAF	BAU
25	PAN	Panama	19.03	29.82	24.53	15.32	12.33	LAM	Base year
26	QAT	Qatar	210.95	29.82	25.94	19.20	17.03	MAF	Actions
27	SAU	Saudi Arabia	861.61	29.82	25.93	19.20	17.03	MAF	Actions
28	ZAF	South Africa	398.00	29.78	25.91	19.18	17.01	MAF	Fixed target
29	ARE	United Arab	473.29	29.73	25.86	19.14	16.98	MAF	Actions
30	EGY	Egypt	438.48	29.71	25.84	19.13	16.97	MAF	Actions
31	IDN	Indonesia	730.88	29.63	24.22	14.87	11.88	Asia	BAU
32	KOR	Korea	239.28	29.54	24.14	14.83	11.84	Asia	BAU
33	PAK	Pakistan	452.92	29.51	24.11	14.81	11.83	Asia	Actions
34	KWT	Kuwait	140.27	29.34	25.52	18.89	16.76	MAF	Actions
35	SGP	Singapore	55.56	29.21	23.87	14.66	11.71	Asia	Intensity
36	THA	Thailand	294.06	29.10	23.78	14.61	11.66	Asia	BAU
37	ETH	Ethiopia	46.12	29.07	25.28	18.72	16.60	MAF	BAU
38	DZA	Algeria	277.24	29.04	25.26	18.70	16.59	MAF	BAU
39	BGD	Bangladesh	204.03	29.03	23.73	14.57	11.64	Asia	BAU
40	ECU	Ecuador	60.74	28.95	23.82	14.87	11.97	LAM	BAU
41	IRQ	Iraq	340.67	28.91	25.15	18.62	16.51	MAF	Fixed BAU
42	PHL	Philippines	257.91	28.81	23.54	14.46	11.55	Asia	BAU
43	VNM	Viet Nam	235.21	28.80	23.53	14.45	11.54	Asia	Base year
44	MYS	Malaysia	488.60	28.79	23.53	14.45	11.54	Asia	Intensity
45	OMN	Oman	111.00	28.66	24.93	18.46	16.37	MAF	Actions
46	COD	Congo	53.29	28.59	24.86	18.41	16.33	MAF	Base year
47	SDN	Sudan	107.64	28.54	24.82	18.38	16.30	MAF	Actions
48	ISR	Israel	78.57	28.44	24.74	18.31	16.24	MAF	Intensity
49	TZA	Tanzania,	70.06	28.36	24.66	18.26	16.20	MAF	Fixed target
50	BHR	Bahrain	46.70	28.32	24.63	18.23	16.17	MAF	Actions
51	KEN	Kenya	95.38	28.27	24.59	18.20	16.14	MAF	Fixed BAU

52 MAR	Morocco	78.86	27.22	23.68	17.53	15.55	MAF	BAU
53 NZL	New Zealand	49.94	25.40	17.62	2.67	-2.28	OBU	Base year
54 GEO	Georgia	9.73	25.36	23.16	19.32	18.08	EES	Intensity
55 DOM	Dominican	34.82	25.16	20.70	12.92	10.41	LAM	Base year
56 BHS	Bahamas	1.78	24.55	20.20	12.61	10.16	LAM	BAU
57 UGA	Uganda	37.90	24.07	20.94	15.50	13.75	MAF	Fixed BAU
58 AGO	Angola	39.57	22.93	19.94	14.76	13.09	MAF	Base year
59 GTM	Guatemala	32.06	21.49	17.68	11.04	8.89	LAM	BAU
60 SUR	Suriname	4.32	21.46	17.66	11.02	8.88	LAM	Actions
61 CRI	Costa Rica	9.05	20.44	16.82	10.50	8.45	LAM	Base year
62 BOL	Bolivia	55.53	19.88	16.36	10.21	8.22	LAM	Actions
63 YEM	Yemen	54.55	19.88	17.29	12.80	11.35	MAF	BAU
64 TUR	Turkey	336.65	19.65	6.13	-17.28	-24.78	OWE	BAU
65 TUN	Tunisia	85.63	18.87	16.41	12.15	10.78	MAF	Intensity
66 TJK	Tajikistan	11.40	18.86	17.22	14.37	13.45	EES	Base year
67 LBN	Lebanon	33.61	18.60	16.18	11.98	10.62	MAF	BAU
68 NOR	Norway	32.69	18.53	5.78	-16.29	-23.37	OWE	Base year
69 CHE	Switzerland	26.80	18.51	5.77	-16.28	-23.34	OWE	Base year
70 ARM	Armenia	17.58	18.13	16.55	13.81	12.92	EES	Fixed target
71 MDA	Moldova	9.80	18.06	16.49	13.76	12.87	EES	Base year
72 PRY	Paraguay	34.32	17.83	14.67	9.16	7.38	LAM	BAU
73 MMR	Myanmar	80.50	17.50	14.30	8.78	7.01	Asia	Actions
74 BRB	Barbados	0.82	17.49	14.39	8.98	7.23	LAM	Base year
75 GHA	Ghana	33.56	17.44	15.17	11.23	9.96	MAF	Fixed BAU
76 KGZ	Kyrgyzstan	19.35	17.22	15.72	13.12	12.28	EES	BAU
77 GNQ	Equatorial	9.39	17.17	14.94	11.06	9.81	MAF	Base year
78 CMR	Cameroon	50.49	15.88	13.81	10.22	9.07	MAF	BAU
79 JOR	Jordan	64.10	15.30	13.31	9.85	8.74	MAF	BAU
80 CIV	Côte d'Ivoire	33.56	14.88	12.94	9.58	8.50	MAF	BAU
81 MDG	Madagascar	46.45	14.78	12.86	9.52	8.44	MAF	BAU
82 MNG	Mongolia	48.30	14.43	11.79	7.24	5.79	Asia	BAU
83 MUS	Mauritius	3.38	14.21	12.36	9.15	8.11	MAF	BAU
84 MOZ	Mozambique	24.50	13.69	11.91	8.82	7.82	MAF	Actions
85 LKA	Sri Lanka	34.67	12.95	10.59	6.50	5.19	Asia	Base year
86 NER	Niger	45.36	12.86	11.19	8.28	7.35	MAF	BAU
87 BWA	Botswana	9.23	12.79	11.12	8.23	7.30	MAF	Base year

88	ZMB	Zambia	16.80	12.65	11.01	8.15	7.23	MAF	BAU
89	HND	Honduras	23.15	12.55	10.32	6.45	5.19	LAM	BAU
90	BFA	Burkina Faso	27.19	12.31	10.71	7.93	7.03	MAF	BAU
91	MLI	Mali	19.72	11.99	10.43	7.72	6.85	MAF	BAU
92	GAB	Gabon	17.87	11.97	10.41	7.71	6.84	MAF	BAU
93	SLV	El Salvador	11.67	11.83	9.73	6.08	4.89	LAM	Actions
94	NIC	Nicaragua	7.92	11.53	9.49	5.92	4.77	LAM	BAU
95	HTI	Haiti	12.15	10.90	8.97	5.60	4.51	LAM	BAU
96	BTN	Bhutan	3.07	10.87	8.88	5.46	4.36	Asia	Actions
97	SRB	Serbia	52.17	10.79	3.36	-9.49	-13.61	OWE	Base year
98	LCA	Saint Lucia	0.39	10.43	8.58	5.36	4.32	LAM	Fixed BAU
99	JAM	Jamaica	12.88	10.37	8.54	5.33	4.29	LAM	BAU
100	VCT	Saint Vincent	0.19	10.27	8.45	5.27	4.25	LAM	BAU
101	MWI	Malawi	15.04	10.19	8.86	6.56	5.82	MAF	Actions
102	SEN	Senegal	27.26	9.95	8.66	6.41	5.69	MAF	BAU
103	ZWE	Zimbabwe	40.58	9.94	8.64	6.40	5.67	MAF	Intensity
104	ISL	Iceland	1.86	9.45	2.95	-8.31	-11.92	OWE	Base year
105	NPL	Nepal	37.16	9.27	7.58	4.65	3.72	Asia	Actions
106	TCD	Chad	21.31	9.24	8.04	5.95	5.28	MAF	BAU
107	COG	Congo	21.13	8.81	7.66	5.67	5.03	MAF	BAU
108	GIN	Guinea	25.74	8.80	7.65	5.67	5.03	MAF	Actions
109	NAM	Namibia	1.21	8.55	7.43	5.50	4.88	MAF	BAU
110	MDV	Maldives	1.21	8.43	6.89	4.23	3.38	Asia	BAU
111	BLZ	Belize	1.18	8.13	6.69	4.17	3.36	LAM	Actions
112	RWA	Rwanda	7.42	7.32	6.37	4.71	4.18	MAF	Actions
113	BEN	Benin	13.34	7.04	6.12	4.53	4.02	MAF	BAU
114	STP	Sao Tome and Principe	0.31	6.83	5.94	4.40	3.90	MAF	Fixed BAU
115	KHM	Cambodia	29.99	6.64	5.43	3.33	2.66	Asia	BAU
116	BIH	Bosnia and	18.14	6.37	1.98	-5.60	-8.03	OWE	BAU
117	LAO	Lao People's	11.26	6.19	5.06	3.11	2.48	Asia	Actions
118	MKD	Macedonia	9.24	5.87	1.83	-5.17	-7.41	OWE	BAU
119	CPV	Cabo Verde	0.85	5.75	5.00	3.70	3.28	MAF	Actions
120	MRT	Mauritania	12.65	5.58	4.86	3.60	3.19	MAF	BAU
121	BDI	Burundi	6.07	5.41	4.71	3.48	3.09	MAF	BAU
122	SWZ	Eswatini	3.13	5.21	4.53	3.35	2.98	MAF	Actions

123	SLE	Sierra Leone	8.26	4.81	4.18	3.09	2.75	MAF	Fixed target
124	TGO	Togo	8.58	4.64	4.04	2.99	2.65	MAF	BAU
125	ALB	Albania	8.64	4.40	1.37	-3.87	-5.55	OWE	BAU
126	FJI	Fiji	1.86	4.35	3.55	2.18	1.74	Asia	BAU
127	MNE	Montenegro	1.79	4.06	1.26	-3.57	-5.12	OWE	Base year
128	CAF	Central	9.73	3.77	3.28	2.42	2.15	MAF	BAU
129	LBR	Liberia	8.28	3.68	3.20	2.37	2.10	MAF	BAU
130	LSO	Lesotho	4.65	3.36	2.92	2.16	1.92	MAF	BAU
131	DJI	Djibouti	1.67	3.25	2.82	2.09	1.85	MAF	BAU
132	GMB	Gambia	1.50	2.57	2.23	1.65	1.47	MAF	BAU
133	GNB	Guinea-Bissau	2.70	2.20	1.91	1.42	1.26	MAF	Actions
134	COM	Comoros	0.12	1.55	1.35	1.00	0.89	MAF	BAU

Note: the negative improved proportion means that there is no required improvement in emission reduction.

Supplementary Table 3| Improved actions for achieving the 1.5 °C target for each country.

			$MtCO_2$-eq	Improved proportion (%)						
No.	ISO	Country	Current NDC	SP1.5A	SP1.5B	SP1.5C	SP1.5D	SP1.5E	Region	NDC Type
1	JPN	Japan	1019.97	102.21	99.28	101.02	101.72	99.86	JPN	Base year
2	USA	United States	5096.13	95.62	90.74	91.64	94.37	90.97	USA	Base year
3	RUS	Russian	2265.35	87.34	84.65	85.25	85.45	83.85	RUS	Base year
4	EU	EU	3409.51	75.21	70.49	71.62	71.37	68.95	EU	Base year
5	CHN	China	13288.86	67.08	64.90	65.29	65.17	64.11	CHN	Intensity
6	CAN	Canada	520.41	66.09	64.69	66.17	66.19	63.63	OBU	Base year
7	AUS	Australia	411.95	65.72	64.33	65.80	65.82	63.27	OBU	Base year
8	IND	India	8071.76	60.16	59.39	59.58	59.57	59.20	IND	Intensity
9	UKR	Ukraine	527.69	59.23	56.87	57.30	57.16	56.01	EES	Base year
10	KAZ	Kazakhstan	318.11	59.17	56.81	57.23	57.10	55.94	EES	Base year
11	UZB	Uzbekistan	491.87	58.62	56.28	56.70	56.57	55.42	EES	Intensity
12	BLR	Belarus	64.16	57.88	55.57	55.99	55.86	54.73	EES	Base year
13	BRA	Brazil	633.18	49.41	45.44	46.08	45.86	43.93	LAM	Base year
14	MEX	Mexico	638.21	49.03	45.09	45.73	45.51	43.60	LAM	BAU
15	ARG	Argentina	483.00	47.91	44.05	44.68	44.47	42.59	LAM	BAU
16	VEN	Venezuela,	362.52	47.02	43.24	43.85	43.65	41.81	LAM	BAU
17	COL	Colombia	179.97	46.96	43.18	43.79	43.59	41.75	LAM	BAU

18	CHL	Chile	185.69	46.43	42.69	43.30	43.09	41.28	LAM	Intensity
19	PER	Peru	93.67	46.27	42.55	43.15	42.95	41.14	LAM	BAU
20	AZE	Azerbaijan	60.60	42.10	40.42	40.73	40.63	39.81	EES	Base year
21	TTO	Trinidad and Tobago	28.96	35.72	32.85	33.32	33.16	31.76	LAM	BAU
22	URY	Uruguay	36.17	31.61	29.07	29.48	29.34	28.10	LAM	Actions
23	PAN	Panama	19.03	31.12	28.62	29.02	28.88	27.67	LAM	Base year
24	IDN	Indonesia	730.88	31.00	28.45	28.88	28.69	27.46	Asia	BAU
25	KOR	Korea	239.28	30.91	28.36	28.79	28.60	27.37	Asia	BAU
26	PAK	Pakistan	452.92	30.87	28.33	28.76	28.57	27.34	Asia	Actions
27	NGA	Nigeria	610.31	30.86	29.02	29.37	29.25	28.32	MAF	BAU
28	IRN	Iran	826.85	30.86	29.02	29.37	29.25	28.32	MAF	BAU
29	QAT	Qatar	210.95	30.86	29.02	29.37	29.25	28.31	MAF	Actions
30	SAU	Saudi Arabia	861.61	30.86	29.02	29.36	29.25	28.31	MAF	Actions
31	ZAF	South Africa	398.00	30.82	28.98	29.33	29.22	28.28	MAF	Fixed target
32	ARE	United Arab	473.29	30.77	28.93	29.28	29.16	28.23	MAF	Actions
33	EGY	Egypt	438.48	30.75	28.92	29.26	29.15	28.21	MAF	Actions
34	SGP	Singapore	55.56	30.56	28.04	28.47	28.28	27.06	Asia	Intensity
35	THA	Thailand	294.06	30.44	27.94	28.36	28.18	26.96	Asia	BAU
36	BGD	Bangladesh	204.03	30.37	27.87	28.29	28.11	26.90	Asia	BAU
37	KWT	Kuwait	140.27	30.36	28.55	28.89	28.78	27.86	MAF	Actions
38	ECU	Ecuador	60.74	30.22	27.79	28.18	28.04	26.86	LAM	BAU
39	PHL	Philippines	257.91	30.14	27.66	28.07	27.89	26.69	Asia	BAU
40	VNM	Viet Nam	235.21	30.13	27.65	28.06	27.88	26.68	Asia	Base year
41	MYS	Malaysia	488.60	30.12	27.64	28.06	27.88	26.68	Asia	Intensity
42	ETH	Ethiopia	46.12	30.08	28.29	28.63	28.51	27.60	MAF	BAU
43	DZA	Algeria	277.24	30.06	28.26	28.60	28.49	27.58	MAF	BAU
44	IRQ	Iraq	340.67	29.92	28.14	28.48	28.36	27.45	MAF	Fixed BAU
45	OMN	Oman	111.00	29.67	27.90	28.23	28.12	27.22	MAF	Actions
46	COD	Congo	53.29	29.58	27.82	28.15	28.04	27.14	MAF	Base year
47	SDN	Sudan	107.64	29.54	27.77	28.11	28.00	27.10	MAF	Actions
48	ISR	Israel	78.57	29.44	27.68	28.01	27.90	27.01	MAF	Intensity
49	TZA	Tanzania	70.06	29.35	27.60	27.93	27.82	26.93	MAF	Fixed target
50	BHR	Bahrain	46.70	29.31	27.56	27.89	27.78	26.89	MAF	Actions
51	KEN	Kenya	95.38	29.26	27.51	27.84	27.73	26.84	MAF	Fixed BAU
52	MAR	Morocco	78.86	28.17	26.49	26.81	26.70	25.85	MAF	BAU

53	DOM	Dominican	34.82	26.26	24.15	24.49	24.37	23.35	LAM	Base year
54	GEO	Georgia	9.73	25.93	24.89	25.08	25.02	24.52	EES	Intensity
55	BHS	Bahamas	1.78	25.62	23.56	23.90	23.78	22.78	LAM	BAU
56	NZL	New Zealand	49.94	25.40	24.86	25.43	25.44	24.45	OBU	Base year
57	UGA	Uganda	37.90	24.91	23.43	23.71	23.61	22.86	MAF	Fixed BAU
58	AGO	Angola	39.57	23.73	22.31	22.58	22.49	21.77	MAF	Base year
59	TUR	Turkey	336.65	23.21	16.87	18.05	17.72	14.65	OWE	BAU
60	GTM	Guatemala	32.06	22.43	20.62	20.92	20.82	19.94	LAM	BAU
61	SUR	Suriname	4.32	22.40	20.59	20.88	20.79	19.91	LAM	Actions
62	NOR	Norway	32.69	21.89	15.90	17.02	16.71	13.81	OWE	Base year
63	CHE	Switzerland	26.80	21.87	15.89	17.00	16.69	13.79	OWE	Base year
64	CRI	Costa Rica	9.05	21.33	19.62	19.89	19.80	18.97	LAM	Base year
65	BOL	Bolivia	55.53	20.75	19.08	19.35	19.26	18.45	LAM	Actions
66	YEM	Yemen	54.55	20.57	19.35	19.58	19.50	18.88	MAF	BAU
67	TUN	Tunisia	85.63	19.53	18.36	18.59	18.51	17.92	MAF	Intensity
68	TJK	Tajikistan	11.40	19.28	18.51	18.65	18.61	18.23	EES	Base year
69	LBN	Lebanon	33.61	19.25	18.10	18.32	18.25	17.66	MAF	BAU
70	PRY	Paraguay	34.32	18.61	17.11	17.36	17.27	16.55	LAM	BAU
71	ARM	Armenia	17.58	18.53	17.79	17.92	17.88	17.52	EES	Fixed target
72	MDA	Moldova	9.80	18.46	17.72	17.86	17.81	17.45	EES	Base year
73	MMR	Myanmar	80.50	18.31	16.80	17.05	16.94	16.21	Asia	Actions
74	BRB	Barbados	0.82	18.26	16.79	17.02	16.94	16.23	LAM	Base year
75	GHA	Ghana	33.56	18.05	16.97	17.17	17.11	16.56	MAF	Fixed BAU
76	GNQ	Equatorial	9.39	17.77	16.71	16.91	16.84	16.30	MAF	Base year
77	KGZ	Kyrgyzstan	19.35	17.60	16.90	17.03	16.99	16.64	EES	BAU
78	CMR	Cameroon	50.49	16.43	15.45	15.64	15.57	15.08	MAF	BAU
79	JOR	Jordan	64.10	15.84	14.89	15.07	15.01	14.53	MAF	BAU
80	CIV	Côte d'Ivoire	33.56	15.40	14.48	14.65	14.59	14.13	MAF	BAU
81	MDG	Madagascar	46.45	15.30	14.39	14.56	14.50	14.04	MAF	BAU
82	MNG	Mongolia	48.30	15.10	13.86	14.06	13.98	13.37	Asia	BAU
83	MUS	Mauritius	3.38	14.70	13.83	13.99	13.94	13.49	MAF	BAU
84	MOZ	Mozambique	24.50	14.17	13.32	13.48	13.43	13.00	MAF	Actions
85	LKA	Sri Lanka	34.67	13.55	12.44	12.62	12.54	12.00	Asia	Base year
86	NER	Niger	45.36	13.31	12.52	12.67	12.62	12.22	MAF	BAU
87	BWA	Botswana	9.23	13.23	12.44	12.59	12.54	12.14	MAF	Base year
88	HND	Honduras	23.15	13.10	12.04	12.21	12.16	11.64	LAM	BAU

89	ZMB	Zambia	16.80	13.10	12.31	12.46	12.41	12.02	MAF	BAU
90	SRB	Serbia	52.17	12.75	9.26	9.91	9.73	8.04	OWE	Base year
91	BFA	Burkina Faso	27.19	12.74	11.98	12.12	12.07	11.69	MAF	BAU
92	MLI	Mali	19.72	12.41	11.67	11.81	11.77	11.39	MAF	BAU
93	GAB	Gabon	17.87	12.39	11.65	11.79	11.74	11.37	MAF	BAU
94	SLV	El Salvador	11.67	12.34	11.35	11.51	11.46	10.98	LAM	Actions
95	NIC	Nicaragua	7.92	12.03	11.07	11.22	11.17	10.70	LAM	BAU
96	HTI	Haiti	12.15	11.38	10.47	10.61	10.56	10.12	LAM	BAU
97	BTN	Bhutan	3.07	11.37	10.43	10.59	10.52	10.07	Asia	Actions
98	ISL	Iceland	1.86	11.17	8.11	8.68	8.53	7.05	OWE	Base year
99	LCA	Saint Lucia	0.39	10.89	10.01	10.15	10.11	9.68	LAM	Fixed BAU
100	JAM	Jamaica	12.88	10.83	9.96	10.10	10.05	9.63	LAM	BAU
101	VCT	Saint Vincent	0.19	10.72	9.85	9.99	9.95	9.53	LAM	BAU
102	MWI	Malawi	15.04	10.55	9.92	10.04	10.00	9.68	MAF	Actions
103	SEN	Senegal	27.26	10.30	9.69	9.80	9.77	9.45	MAF	BAU
104	ZWE	Zimbabwe	40.58	10.28	9.67	9.79	9.75	9.43	MAF	Intensity
105	NPL	Nepal	37.16	9.70	8.90	9.04	8.98	8.59	Asia	Actions
106	TCD	Chad	21.31	9.57	9.00	9.10	9.07	8.78	MAF	BAU
107	COG	Congo	21.13	9.12	8.58	8.68	8.64	8.37	MAF	BAU
108	GIN	Guinea	25.74	9.11	8.56	8.67	8.63	8.36	MAF	Actions
109	NAM	Namibia	1.21	8.85	8.32	8.42	8.38	8.12	MAF	BAU
110	MDV	Maldives	1.21	8.82	8.10	8.22	8.17	7.81	Asia	BAU
111	BLZ	Belize	1.18	8.48	7.80	7.91	7.87	7.54	LAM	Actions
112	RWA	Rwanda	7.42	7.58	7.12	7.21	7.18	6.95	MAF	Actions
113	BIH	Bosnia and	18.14	7.52	5.47	5.85	5.74	4.75	OWE	BAU
114	BEN	Benin	13.34	7.29	6.85	6.93	6.91	6.68	MAF	BAU
115	STP	Sao Tome and Principe	0.31	7.07	6.65	6.73	6.70	6.48	MAF	Fixed BAU
116	KHM	Cambodia	29.99	6.95	6.37	6.47	6.43	6.15	Asia	BAU
117	MKD	Macedonia	9.24	6.94	5.04	5.40	5.30	4.38	OWE	BAU
118	LAO	Lao Republic	11.26	6.47	5.94	6.03	5.99	5.73	Asia	Actions
119	CPV	Cabo Verde	0.85	5.95	5.59	5.66	5.64	5.46	MAF	Actions
120	MRT	Mauritania	12.65	5.78	5.43	5.50	5.48	5.30	MAF	BAU
121	BDI	Burundi	6.07	5.60	5.26	5.33	5.31	5.14	MAF	BAU
122	SWZ	Eswatini	3.13	5.39	5.07	5.13	5.11	4.95	MAF	Actions
123	ALB	Albania	8.64	5.19	3.77	4.04	3.96	3.28	OWE	BAU

124	SLE	Sierra Leone	8.26	4.97	4.68	4.73	4.72	4.56	MAF	Fixed target
125	TGO	Togo	8.58	4.80	4.52	4.57	4.55	4.41	MAF	BAU
126	MNE	Montenegro	1.79	4.79	3.48	3.73	3.66	3.02	OWE	Base year
127	FJI	Fiji	1.86	4.55	4.18	4.24	4.21	4.03	Asia	BAU
128	CAF	Central	9.73	3.90	3.66	3.71	3.69	3.58	MAF	BAU
129	LBR	Liberia	8.28	3.81	3.58	3.63	3.61	3.50	MAF	BAU
130	LSO	Lesotho	4.65	3.48	3.27	3.31	3.30	3.19	MAF	BAU
131	DJI	Djibouti	1.67	3.36	3.16	3.20	3.19	3.08	MAF	BAU
132	GMB	Gambia	1.50	2.66	2.50	2.53	2.52	2.44	MAF	BAU
133	GNB	Guinea-Bissau	2.70	2.28	2.14	2.17	2.16	2.09	MAF	Actions
134	COM	Comoros	0.12	1.61	1.51	1.53	1.52	1.47	MAF	BAU

Note: the negative improved proportion means that there is no required improvement in emission reduction.
