# Supplementary material for: Self-preservation strategy for approaching global warming targets in the post-Paris Agreement era
Source: Nat Commun. 2020 Apr 14;11:1624. doi: 10.1038/s41467-020-15453-z (PMC7156390; doi:10.1038/s41467-020-15453-z)
Supplement: Supplementary file 3 — Reporting Summary [file 41467_2020_15453_MOESM3_ESM.pdf]

nature research

Corresponding author(s): DBPR NCOMMS-19-28971C

Last updated by author(s): Mar 6, 2020

Reporting Summary

Nature Research wishes to improve the reproducibility of the work that we publish. This form provides structure for consistency and transparency in reporting. For further information on Nature Research policies, see Authors & Referees and the Editorial Policy Checklist.

Statistics

For all statistical analyses, confirm that the following items are present in the figure legend, table legend, main text, or Methods section.

n/a	Confirmed	
☒	☐	The exact sample size (*n*) for each experimental group/condition, given as a discrete number and unit of measurement
☒	☐	A statement on whether measurements were taken from distinct samples or whether the same sample was measured repeatedly
☒	☐	The statistical test(s) used AND whether they are one- or two-sided<br>*Only common tests should be described solely by name; describe more complex techniques in the Methods section.*
☒	☐	A description of all covariates tested
☒	☐	A description of any assumptions or corrections, such as tests of normality and adjustment for multiple comparisons
☒	☐	A full description of the statistical parameters including central tendency (e.g. means) or other basic estimates (e.g. regression coefficient) AND variation (e.g. standard deviation) or associated estimates of uncertainty (e.g. confidence intervals)
☒	☐	For null hypothesis testing, the test statistic (e.g. *F*, *t*, *r*) with confidence intervals, effect sizes, degrees of freedom and *P* value noted<br>*Give P values as exact values whenever suitable.*
☒	☐	For Bayesian analysis, information on the choice of priors and Markov chain Monte Carlo settings
☒	☐	For hierarchical and complex designs, identification of the appropriate level for tests and full reporting of outcomes
☒	☐	Estimates of effect sizes (e.g. Cohen's *d*, Pearson's *r*), indicating how they were calculated

Our web collection on statistics for biologists contains articles on many of the points above.

Software and code

Policy information about availability of computer code

Data collection	This study used Matlab R2018b to do data pre-processing.
Data analysis	This study used Stata 15.1 to project the greenhouse gases emission intensity and GAMS 24.8.5 to simulate the win-win strategy.

For manuscripts utilizing custom algorithms or software that are central to the research but not yet described in published literature, software must be made available to editors/reviewers. We strongly encourage code deposition in a community repository (e.g. GitHub). See the Nature Research guidelines for submitting code & software for further information.

Data

Policy information about availability of data

All manuscripts must include a data availability statement. This statement should provide the following information, where applicable:

- Accession codes, unique identifiers, or web links for publicly available datasets
- A list of figures that have associated raw data
- A description of any restrictions on data availability

The data that support the plots within this paper and the findings of this study are available from the corresponding author upon reasonable request. Authors provide a list of figures that have associated raw data. Requests for code will be jointly considered by the corresponding authors .

Field-specific reporting

Please select the one below that is the best fit for your research. If you are not sure, read the appropriate sections before making your selection.

☐ Life sciences ☐ Behavioural & social sciences ☒ Ecological, evolutionary & environmental sciences

Ecological, evolutionary & environmental sciences study design

All studies must disclose on these points even when the disclosure is negative.

Study description	This study presents a self-preservation strategy of current climate pledges that can achieve the well below 2°C or 1.5°C target and meanwhile generate economic benefit for all ratified parties compared with current NDCs, by considering the uncertainties of climate damage, technology development and equity principles. The panel data were used to forecast the emission intensities first and then the China's Climate Change Integrated Assessment Model (C3IAM) was employed to optimize and simulate the win-win strategies. A better emission-reduction strategy than current NDCs in terms of the potential net income from climate mitigation would be more informative for countries to reset their goals and update their NDCs in the post-Paris Agreement era.
Research sample	For the historical social-economic and emissions data, this study used GDP from PWT9.0, population from UN, CO2 emission from CDIAC and UNFCCC, CH4 and N2O emissions from EDGAR. For the future data, this study used GDP from SSP2 and population from UN. For the simulation of the win-win strategy, additional sources (IMF) was applied.
Sampling strategy	This study used the relevant socio-economic and emissions data for all the countries that have submitted the NDC. So it is a full sample.
Data collection	This study obtained national raw socio-economic and emissions data from the public source and aggregate the national data to the regional data for simulation.
Timing and spatial scale	As for the projection section, this study considered the panel data for 182 economic entities over the period 1950-2014 to project the emission intensities from 2015 to 2100. As for simulation section, this study considered more than 200 years (45 periods with 5 year step length) simulation for 12 regions or countries from 2010.
Data exclusions	This study excluded the countries who did not submit national determined contributions (NDC).
Reproducibility	This study did not include any experiment. Regarding the method, this study solved the optimization problem using GAMS solver in the simulation section.
Randomization	Randomization is not relevant to this study, because this study only used the statistical raw data for projection and optimization.
Blinding	Blinding is not relevant to this study, because there is no experiment in this study.

Did the study involve field work? ☐ Yes ☒ No

Reporting for specific materials, systems and methods

We require information from authors about some types of materials, experimental systems and methods used in many studies. Here, indicate whether each material, system or method listed is relevant to your study. If you are not sure if a list item applies to your research, read the appropriate section before selecting a response.

Materials & experimental systems

n/a	Involved in the study
☒	☐ Antibodies
☒	☐ Eukaryotic cell lines
☒	☐ Palaeontology
☒	☐ Animals and other organisms
☒	☐ Human research participants
☒	☐ Clinical data

Methods

n/a	Involved in the study
☒	☐ ChIP-seq
☒	☐ Flow cytometry
☒	☐ MRI-based neuroimaging
